# Performance comparison of gadoxetic and extracellular MRI in diagnosing subcentimeter recurrent HCC after hepatectomy: a modified algorithm

**DOI:** 10.1186/s13244-026-02266-9

**Published:** 2026-04-08

**Authors:** Yuyao Xiao, Fei Wu, Changwu Zhou, Peng Huang, Cheng Wang, Haoran Dai, Xinyue Liang, Xi Jia, Mengsu Zeng

**Affiliations:** 1 Shanghai Institute of Medical Imaging, Shanghai, China; 2 Department of Radiology, Zhongshan Hospital, Fudan University, Shanghai, China; 3 Department of Radiology, The Affiliated Hospital of Yangzhou University, Yangzhou University, Yangzhou, China; 4 Department of Cancer Center, Zhongshan Hospital, Fudan University, Shanghai, China

**Keywords:** Liver neoplasms, Magnetic resonance imaging, Diagnosis criteria

## Abstract

**Objectives:**

To compare the performance of gadoxetic acid-enhanced MRI (EOB-MRI) and extracellular contrast agent-enhanced MRI (ECA-MRI) for diagnosing subcentimeter recurrent HCC.

**Materials and methods:**

Between January 2017 and December 2023, patients with newly detected suspicious subcentimeter hepatic lesions who underwent both EOB-MRI and ECA-MRI after HCC hepatectomy were retrospectively and consecutively included and divided into training and time-independent test sets. The generalized estimating equation model identified significant MR characteristics in the training set. Diagnostic performances of typical vascular pattern and modified diagnostic algorithms were calculated and compared using the McNemar test in both sets.

**Results:**

A total of 153 patients (mean age, 55.30 ± 11.09; 118 men) with 185 subcentimeter lesions were included. Typical vascular pattern exhibited comparable but unsatisfactory performance across modalities (both sensitivity < 0.50). In the training set, the optimal modified algorithms for EOB-MRI and ECA-MRI combined all identified significant MR characteristics: T2WI mild-to-moderate hyperintensity, nonperipheral washout (on PVP/TP for EOB-MRI; on PVP/DP for ECA-MRI), and restricted diffusion. Both modified algorithms maintained a comparable high specificity compared to the typical vascular pattern. However, the EOB-MRI-based algorithm demonstrated significantly higher sensitivity than the typical vascular pattern (training set, 0.812 vs. 0.400, *p* < 0.001; time-independent test set, 0.792 vs. 0.375, *p* = 0.006), an improvement not observed with the ECA-MRI-based algorithm. Furthermore, EOB-MRI-based modified algorithm demonstrated higher sensitivity than its ECA-MRI counterpart (training set, 0.812 vs. 0.425, *p* < 0.001; time-independent test set, 0.792 vs. 0.417, *p* = 0.004), with comparable specificity.

**Conclusion:**

EOB-MRI with a modified algorithm exhibited superior diagnostic performance for subcentimeter recurrent HCC when compared with ECA-MRI.

**Critical relevance statement:**

This study provides evidence comparing gadoxetic and extracellular MRI in diagnosing subcentimeter recurrent HCC, highlighting the superior diagnostic performance of EOB-MRI-based modified algorithm and its potential to optimize surveillance strategies and clinical decision-making.

**Key Points:**

- Early detection of subcentimeter recurrent HCC optimizes treatment windows.
- EOB-MRI-based modified algorithm improves sensitivity while maintaining high specificity.
- EOB-MRI-based modified algorithm expedites diagnosing subcentimeter recurrences, ultimately facilitating salvage treatment.

**Graphical Abstract:**

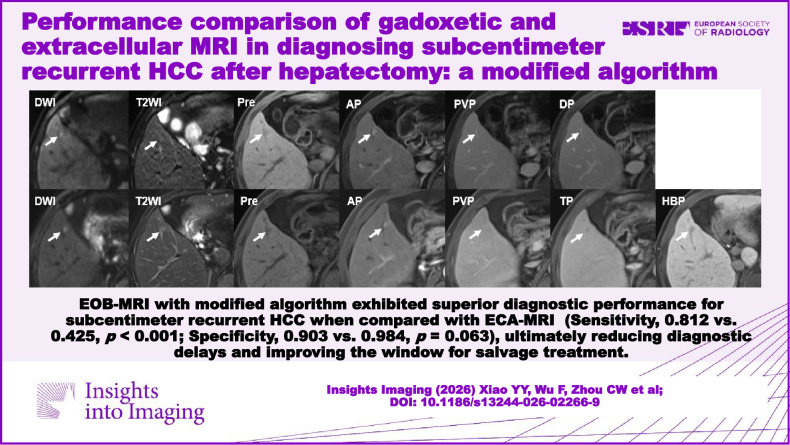

## Introduction

As the primary curative modality for hepatocellular carcinoma (HCC), hepatectomy’s long-term effectiveness remains challenged by a > 70% recurrence rate within 5 years [1, 2], critically impacting clinical outcomes. Repeat hepatectomy, salvage liver transplantation, systemic treatment and some locoregional therapies were used to treat recurrent HCC. Several researchers have proposed minimally invasive therapies for subcentimeter recurrent HCC as soon as possible in patients with a prior history of HCC [3], and Song et al [4] also have demonstrated that percutaneous US/MRI fusion-guided radiofrequency ablation has been proved a safe and effective treatment modality for patients with subcentimeter recurrent HCC (≤ 1 cm), which emphasized the need to identify early recurrences before tumor progression compromises therapeutic windows.

According to guidelines from the American Association for the Study of Liver Diseases (AASLD) and the European Association for the Study of the Liver (EASL), HCC can be noninvasively diagnosed based on its typical vascular pattern, that is, arterial phase hyperenhancement followed by washout, which has also been widely validated in the context of recurrent HCC [5–7]. Furthermore, the AASLD has adopted the Liver Imaging Reporting and Data System (LI-RADS), recognizing its superior accuracy in stratifying the likelihood of HCC [8]. As these guidelines prioritize high specificity, they do not permit a defi nitive diagnosis of subcentimeter recurrent HCC; therefore, a wait-and-see strategy is recommended. However, this strategy may be suboptimal for suspicious subcentimeter recurrences after HCC hepatectomy, as biologically aggressive subcentimeter recurrences can progress rapidly [9]. The absence of an optimal imaging modality and diagnostic algorithm further complicates clinical decision-making.

Gadoxetic acid-enhanced MRI (EOB-MRI) integrates dynamic contrast phases similar to extracellular contrast agent-enhanced MRI (ECA-MRI) with an additional hepatobiliary phase (HBP) imaging [10]. The advantages of HBP in improving the detection accuracy of small lesions and providing valuable information for diagnosing HCC have been well recognized [11–13]. Moreover, previous studies suggest that including the transitional phase (TP) in the washout assessment can improve sensitivity for diagnosing HCC without compromising specificity [14, 15], even in subcentimeter HCC [16]. Nonetheless, EOB-MRI has also recognized limitations in depicting key imaging features such as arterial phase hyperenhancement, washout, and enhancing capsule-major features for HCC diagnosis according to LI-RADS [17–19]. Therefore, understanding discrepancies between EOB-MRI and ECA-MRI in diagnosing subcentimeter recurrent HCC is essential.

Therefore, this study aims to compare the performance of EOB-MRI and ECA-MRI in diagnosing subcentimeter recurrent HCC, in an effort to provide evidence-based support for selecting appropriate follow-up tools for patients after HCC hepatectomy.

## Materials and methods

### Patient selection

**Fig. 1:**
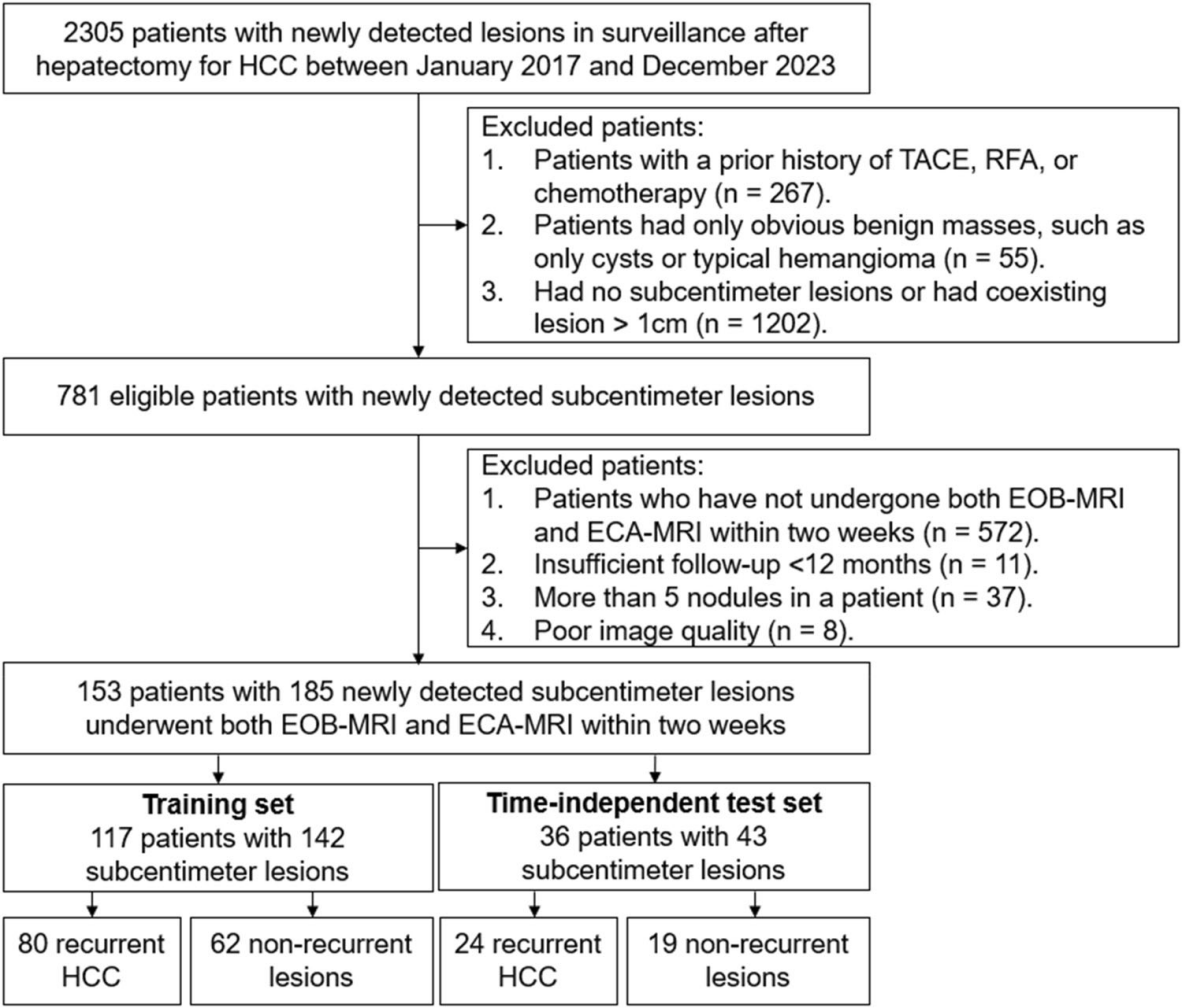
Flowchart of the study. HCC, hepatocellular carcinoma; EOB-MRI, gadoxetate acid-enhanced MRI; ECA-MRI, extracellular contrast agent-enhanced MRI

Our retrospective study was approved by the institutional review board of Zhongshan Hospital, Fudan University (B2021-325R), and was in accordance with the Declaration of Helsinki, with the requirement for informed consent being waived. Between January 2017 and December 2023, 2305 patients with newly detected lesions in surveillance (by using ultrasound, CT or MRI) after HCC hepatectomy were initially included in our study. 1524 patients were excluded due to: (1) patients with a prior history of TACE, RFA, or chemotherapy; (2) obvious benign lesions (e.g., cysts or typical hemangiomas); and (3) no subcentimeter lesions or had coexisting lesions > 1 cm. In the remaining 781 eligible patients with suspicious subcentimeter lesions, another 628 patients were excluded due to: (1) patients who have not undergone both EOB-MRI and ECA-MRI within 2 weeks; (2) number of lesions > 5; (3) insufficient follow-up < 12 months; (4) poor image quality. The flowchart of inclusion is presented in Fig. 1. Finally, 153 patients after HCC hepatectomy with 185 subcentimeter suspicious hepatic lesions, all of whom underwent both EOB-MRI and ECA-MRI within 2 weeks, were included in our study. They were divided into two cohorts: a training set, which was used to develop and optimize diagnostic algorithms (117 patients with 142 lesions from January 2017 to June 2022), and a time-independent test set, which was used to validate the diagnostic performance of diagnostic algorithms in a temporally independent way (36 patients with 43 lesions from July 2022 to December 2023).

### MR examinations

MR images were acquired via a 3.0-T MR scanner (MAGNETOM Prisma, Siemens Healthineers). Routine plain-scan sequences included T1-weighted in-phase and out-of-phase imaging, transverse T2-weighted fast spin-echo, and diffusion-weighted imaging (DWI, b = 0, 50, and 500 s/mm^2^).

Dynamic imaging was performed with a T1-weighted fat-suppressed sequence. For ECA-MRI, Gadobutrol (Gadavist; Bayer HealthCare) was intravenously administered (0.1 mmol/kg, 2 mL/s). The arterial phase (AP) was triggered when the contrast agent reached the ascending aorta, followed by the portal venous phase (PVP, 60 s) and delayed phase (DP, 3 min). For EOB-MRI, gadoxetate disodium (Primovist, Bayer HealthCare) was intravenously administered (0.025 mmol/kg, 1 mL/s). AP was similarly triggered, followed by portal venous phase (PVP, 60 s), transitional phase (TP; 3 min), and hepatobiliary phase (HBP; 20 min) acquisitions.

### Reference standard

Diagnosis of HCC and non-HCC malignancies was confirmed by histopathology via hepatectomy, transplantation, or percutaneous biopsy. Benign lesions were diagnosed either pathologically or by clinical follow-up. All patients underwent follow-up imaging for ≥ 12 months. Benign diagnoses were assigned based on lesion stability or reduced conspicuity during follow-up when histology was unavailable.

### Image analysis

Two board-certified abdominal radiologists (Y.Y.X. with 15 years of experience, M.S.Z. with 35 years of experience) independently reviewed all images, blinded to patients’ clinical histories and final diagnoses. Each radiologist performed image analysis in two separate sessions, each dedicated to either EOB-MRI or ECA-MRI images. In the first session, either ECA-MRI or EOB-MRI images were randomly assigned. The remaining modality was assessed in the second session, also in a randomized manner. To reduce potential recall or learning bias, a time interval of 4 weeks was maintained between the two sessions. Discordant cases in terms of MR characteristics between the two observers were subsequently resolved by consensus.

On ECA-MRI, the following MR characteristics were evaluated according to explanation detailed in Supplementary Table S1: (1) T1WI hypointensity; (2) T2WI mild-moderate hyperintensity; (3) fat deposition; (4) nonrim arterial phase hyperenhancement (APHE); (5) nonperipheral washout on PVP or DP; (6) enhancing capsule; and (7) restricted diffusion.

On EOB-MRI, following MR characteristics were assessed: (1) T1WI hypointensity; (2) T2WI mild-moderate hyperintensity; (3) fat deposition; (4) nonrim APHE; (5) nonperipheral washout on PVP; (6) TP hypointensity; (7) nonperipheral washout on PVP or TP; (8) HBP hypointensity; (9) enhancing capsule; and (10) restricted diffusion.

Moreover, LI-RADS categorization was also evaluated according to the imaging features mentioned above, according to LI-RADS ver. 2018.

### Statistical analysis

On a per-lesion basis, a generalized estimating equation model using logistic regression with an exchangeable correlation matrix was applied in training set to identify significant MR characteristics while accounting for clustering in patients with multiple lesions. Variance inflation factor (VIF) was used to assess multicollinearity, and a VIF greater than 2 indicates the presence of multicollinearity [20]. MR characteristics with *p*-value < 0.05 in the univariate analysis and no multicollinearity were entered into the multivariate analysis. Odds ratio (OR), 95% confidence interval (CI), and two-sided *p*-value were then reported. In both training and time-independent test sets, diagnostic performance parameters, including sensitivity, specificity, accuracy, positive predictive value (PPV), and negative predictive value (NPV), were calculated and subsequently compared using the McNemar test between EOB-MRI and ECA-MRI. Interobserver agreement on MR characteristics was assessed with κ statistics: ≤ 0.20 (poor), 0.21–0.40 (fair), 0.41–0.60 (moderate), 0.61–0.80 (substantial), and 0.81–1.00 (almost perfect).

Group comparisons between the training and time-independent test sets used independent-sample *t*-tests, chi-square, or Fisher’s exact tests, as appropriate.

All statistical analyses were conducted in R software (version 4.4.2) and SPSS 20.0 (IBM), and the significance level was considered to be a *p*-value < 0.05.

## Results

### Clinicopathologic characteristics of patients and hepatic lesions

A total of 153 patients (mean age, 55.30 ± 11.09; 118 men) with 185 subcentimeter lesions were included. The training set consisted of 117 patients with 142 lesions, and the time-independent test set included 36 patients with 43 lesions. In the training set, 92 patients underwent ECA-MRI before EOB-MRI, while 25 underwent EOB-MRI before ECA-MRI. In the time-independent test set, 22 patients underwent ECA-MRI before EOB-MRI, and 14 underwent EOB-MRI before ECA-MRI. Clinicopathologic characteristics of patients and hepatic lesions in the training set and time-independent test set were detailed in Table 1. There were no significant differences in age, sex, or chronic liver disease conditions between patients in the training set and time-independent test set, as lesion size and distribution of final diagnosis also showed no statistical differences between the two sets of lesions (all *p* > 0.05).

**Table 1:** Clinicopathologic characteristics of patients with newly detected subcentimeter suspicious lesions

Variable	Training set	Test set	*p*-value
Patients	*n* = 117	*n* = 36	
Age, years	55.77 ± 10.71 (range, 29–78)	53.78 ± 12.30 (range, 29–72)	0.190
Male/female	91/26	27/9	0.652
Etiology			> 0.999
Hepatitis B	108 (92.3%)	31 (86.1%)	
Hepatitis C	3 (2.6%)	1 (2.8%)	
Hepatitis B and C	1 (0.8%)	0 (0%)	
Alcoholic liver disease	2 (1.7%)	2 (5.6%)	
Others	3 (2.6%)	2 (5.6%)	
Liver cirrhosis	65 (55.6%)	20 (55.6%)	0.709
Child-Pugh classification			> 0.999
A	114 (97.4%)	35 (97.2%)	
B	3 (2.6%)	1 (2.8%)	
C	0 (0%)	0 (0%)	
Number of lesions			-
1	95 (81.2%)	31 (86.1%)	
2	19 (16.2%)	3 (8.3%)	
3	3 (2.6%)	2 (5.6%)	
Time interval between histological test and index MRI, days	3 (range, 2–7)	4 (range, 2–9)	
Time interval between EOB-MRI and ECA-MRI, days	5 (range, 1–15)	6 (range, 2–15)	-
**Lesions**	*n* = 142	*n* = 43	
Size, mm	7.8 ± 2.0 (range, 3–10)	8.3 ± 1.9 (range, 3–10)	0.348
Final diagnosis			> 0.999
HCC	80 (56.3%)	24 (55.8%)	
Non-HCC malignancy			
Combined hepatocellular-cholangiocarcinoma	1 (0.7%)	0 (0%)	
Lymphoepithelioma-like carcinoma	1 (0.7%)	0 (0%)	
Benign lesion			
Hemangioma	3 (2.1%)	1 (2.3%)	
Focal nodular hyperplasia	1 (0.7%)	0 (0%)	
Dysplastic nodule	39 (27.5%)	12 (27.9%)	
Arterioportal shunts	17 (12.0%)	6 (14.0%)	

Values are mean ± SD (range), number, number (percentage), or median (range)

*HCC* hepatocellular carcinoma, *EOB-MRI* gadoxetate acid-enhanced MRI, *ECA-MRI* extracellular contrast agent-enhanced MRI

### MR characteristics of hepatic lesions

The comparisons of MR characteristics between subcentimeter recurrent HCCs and non-recurrent hepatic lesions on two MRI modalities are described in Supplementary Table S2.

In the training set, the presence of T1WI hypointensity, T2WI mild-moderate hyperintensity, nonrim APHE, restricted diffusion and LR-4/5 were significantly higher in subcentimeter recurrent HCCs than in non-recurrent hepatic lesions on both EOB-MRI and ECA-MRI (all *p* < 0.05), while fat deposition showed no significant differences. Nonperipheral washout on PVP (52.5% vs. 6.5%, *p* < 0.001) and nonperipheral washout on PVP or DP (43.8% vs. 12.9%, *p* < 0.001) were also more common in subcentimeter recurrent HCCs than in non-recurrent hepatic lesions on EOB-MRI and ECA-MRI, respectively. However, enhancing capsule showed significantly higher prevalence in subcentimeter recurrences than in no recurrences on ECA-MRI (16.3% vs. 1.6%, *p* = 0.004), but not on EOB-MRI (8.8% vs. 1.6%, *p* = 0.138). Additionally, TP hypointensity, nonperipheral washout on PVP or TP and HBP hypointensity, which were MR characteristics being evaluated only on EOB-MRI, were exhibited more commonly in subcentimeter recurrent HCC than in non-recurrent hepatic lesions (all *p* < 0.05).

In the time-independent test set, T2WI mild-moderate hyperintensity, restricted diffusion, nonperipheral washout and LR-4/5 showed the same distribution trend as the training set on both MRI modalities, while T1WI hypointensity (83.3% vs. 57.9%, *p* = 0.065) and nonrim APHE (87.5% vs. 68.4%, *p* = 0.153) showed no significant differences between recurrences and non-recurrences in ECA-MRI. The presence of an enhancing capsule was higher in recurrences than in non-recurrences, but without statistical significance, on both MRI modalities (*p* > 0.05). Moreover, subcentimeter recurrent HCCs showed T1WI hypointensity and nonrim APHE more frequently on ECA-MRI than on EOB-MRI.

### Uni-/multivariate analyses for predictive MR characteristics

For EOB-MRI, the univariate analysis showed that T1WI hypointensity, T2WI mild-moderate hyperintensity, nonrim APHE, nonperipheral washout on PVP, TP hypointensity, nonperipheral washout on PVP or TP, HBP hypointensity and restricted diffusion were predictors of subcentimeter recurrent HCCs. Due to multicollinearity between TP hypointensity (VIF = 4.208) and nonperipheral washout on PVP or TP (VIF = 4.613), nonperipheral washout on PVP or TP with a higher OR value (20.882 vs. 11.667) was selected for further multiple regression analysis. Eventually, T2WI mild-moderate hyperintensity (OR, 7.062; 95% CI, 1.926–28.896; *p* = 0.003), nonperipheral washout on PVP or TP (OR, 4.145; 95% CI, 1.139–15.085; *p* = 0.031) and restricted diffusion (OR, 6.799; 95% CI, 1.996–23.155; *p* = 0.002) were independent predictors for subcentimeter recurrent HCC diagnosis in multivariate analysis.

For ECA-MRI, T1WI hypointensity, T2WI mild-moderate hyperintensity, nonrim APHE, nonperipheral washout on PVP or DP, enhancing capsule and restricted diffusion were of statistical significance in the univariate analysis. According to multivariate regression analysis, T2WI mild-moderate hyperintensity (OR, 5.684; 95% CI, 1.844–17.525; *p* = 0.002), nonperipheral washout on PVP or DP (OR, 3.956; 95% CI, 1.259–12.430; *p* = 0.019) and restricted diffusion (OR, 6.486; 95% CI, 2.357–17.850; *p* < 0.001) were independent significant predictors for subcentimeter recurrent HCC (Table 2).

**Table 2:** Univariate and multivariate analysis of MR characteristics for subcentimeter suspicious lesions on EOB-MRI and ECA-MRI

MR characteristics	Univariate analysis		Multivariate analysis	
	OR (95% CI)	*p*-value	OR (95% CI)	*p*-value
EOB-MRI				
T1WI hypointensity	8.928 (2.467–32.305)	0.001	3.100 (0.611–15.726)	0.172
T2WI mild-moderate hyperintensity	20.769 (7.368–58.545)	< 0.001	7.062 (1.926–28.896)	0.003
Fat deposition	1.838 (0.538–6.275)	0.331		
Nonrim APHE	2.489 (1.248–4.964)	0.010	1.180 (0.379–3.676)	0.775
Nonperipheral washout on PVP	16.026 (5.313–48.346)	< 0.001	4.162 (0.813–21.291)	0.087
TP hypointensity*	11.667 (4.809–28.306)	< 0.001		
Nonperipheral washout on PVP or TP*	20.882 (8.575–50.856)	< 0.001	4.145 (1.139–15.085)	0.031
Enhancing capsule	5.849 (0.700–48.863)	0.103		
HBP hypointensity	18.960 (2.391–150.332)	0.005	7.308 (0.669–79.795)	0.103
Restricted diffusion	19.528 (7.356–51.843)	< 0.001	6.799 (1.996–23.155)	0.002
ECA-MRI				
T1WI hypointensity	4.041 (1.692–9.648)	0.002	2.163 (0.693–6.749)	0.184
T2WI mild-moderate hyperintensity	14.034 (5.320–37.025)	< 0.001	5.684 (1.844–17.525)	0.002
Fat deposition	2.185 (0.555–8.606)	0.264		
Nonrim APHE	3.257 (1.566–6.771)	0.002	1.463 (0.542–3.842)	0.463
Nonperipheral washout on PVP or DP	5.250 (2.213–12.456)	< 0.001	3.956 (1.259–12.430)	0.019
Enhancing capsule	11.836 (1.504–93.172)	0.019	3.565 (0.399–31.858)	0.255
Restricted diffusion	11.667 (4.809–28.306)	< 0.001	6.486 (2.357–17.850)	< 0.001

Values are OR (95% CI)

*EOB-MRI* gadoxetate acid-enhanced MRI, *ECA-MRI* extracellular contrast agent-enhanced MRI

* MR characteristics showed multicollinearity, with variance inflation factor of 4.208 and 4.613, and nonperipheral washout on PVP or TP with a higher OR value was selected for further multiple regression analysis

### Diagnostic performance of diagnostic algorithm

Diagnostic performance of typical vascular pattern, LI-RADS categorization, significant MR characteristics in multivariate analysis and their combinations are detailed in Tables 3 and 4.

**Table 3:** Diagnostic performance of diagnostic algorithms in the training set

MR characteristics	Sensitivity	*p*-value	Specificity	*p*-value	Accuracy	PPV	NPV
EOB-MRI							
Typical vascular pattern	0.400 (0.293–0.507)	-	0.984 (0.953–0.999)	-	0.655 (0.577–0.733)	0.970 (0.911–0.999)	0.560 (0.466–0.653)
(a) T2WI mild-moderate hyperintensity	0.938 (0.884–0.991)	< 0.001	0.581 (0.458–0.703)	< 0.001	0.782 (0.714–0.850)	0.743 (0.657–0.828)	0.878 (0.778–0.978)
(b) Nonperipheral washout on PVP or TP	0.888 (0.818–0.957)	< 0.001	0.726 (0.615–0.837)	< 0.001	0.817 (0.753–0.881)	0.807 (0.724–0.889)	0.833 (0.734–0.933)
(c) Restricted diffusion	0.925 (0.867–0.983)	< 0.001	0.613 (0.492–0.734)	< 0.001	0.789 (0.722–0.856)	0.755 (0.670–0.840)	0.864 (0.762–0.965)
(d) = (a) + (b)	0.838 (0.757–0.918)	< 0.001	0.871 (0.788–0.954)	0.016	0.852 (0.794–0.911)	0.893 (0.823–0.963)	0.806 (0.711–0.901)
(e) = (a) + (c)	0.888 (0.818–0.957)	< 0.001	0.774 (0.670–0.878)	< 0.001	0.838 (0.777–0.899)	0.835 (0.756–0.914)	0.842 (0.747–0.937)
(f) = (b) + (c)	0.838 (0.757–0.918)	< 0.001	0.887 (0.808–0.966)	0.031	0.859 (0.802–0.916)	0.905 (0.839–0.972)	0.809 (0.715–0.902)
(a) + (b) + (c)	0.812 (0.727–0.898)	< 0.001	0.903 (0.830–0.977)	0.063	0.852 (0.794–0.911)	0.915 (0.851–0.980)	0.789 (0.694–0.884)
(d) or (e) or (f)	0.950 (0.902–0.998)	< 0.001	0.726 (0.615–0.837)	< 0.001	0.852 (0.794–0.911)	0.817 (0.739–0.896)	0.918 (0.842–0.995)
ECA-MRI							
Typical vascular pattern	0.362 (0.257–0.468)	-	0.968 (0.924–0.999)	-	0.627 (0.547–0.706)	0.935 (0.849–0.999)	0.541 (0.448–0.633)
(g) T2WI mild-moderate hyperintensity	0.925 (0.867–0.983)	< 0.001	0.532 (0.408–0.656)	< 0.001	0.754 (0.683–0.824)	0.718 (0.632–0.805)	0.846 (0.733–0.959)
(h) Washout	0.438 (0.329–0.546)	0.031	0.871 (0.788–0.954)	0.031	0.627 (0.547–0.706)	0.814 (0.698–0.930)	0.545 (0.447–0.644)
(i) Restricted diffusion	0.900 (0.834–0.966)	< 0.001	0.565 (0.441–0.688)	< 0.001	0.754 (0.683–0.824)	0.727 (0.640–0.815)	0.814 (0.698–0.930)
(j) = (g) + (h)	0.425 (0.317–0.533)	0.063	0.952 (0.898–0.999)	> 0.999	0.655 (0.577–0.733)	0.919 (0.831–0.999)	0.562 (0.467–0.657)
(k) = (g) + (i)	0.862 (0.787–0.938)	< 0.001	0.774 (0.670–0.878)	0.004	0.824 (0.761–0.887)	0.831 (0.751–0.912)	0.814 (0.714–0.913)
(l) = (h) + (i)	0.425 (0.317–0.533)	0.063	0.968 (0.924–0.999)	> 0.999	0.662 (0.584–0.740)	0.944 (0.870–0.999)	0.566 (0.472–0.660)
(g) + (h) + (i)	0.425 (0.317–0.533)	0.063	0.984 (0.953–0.999)	> 0.999	0.669 (0.592–0.746)	0.971 (0.916–0.999)	0.570 (0.476–0.664)
(j) or (k) or (l)	0.862 (0.787–0.938)	< 0.001	0.726 (0.615–0.837)	< 0.001	0.803 (0.737–0.868)	0.802 (0.718–0.886)	0.804 (0.700–0.908)

Values in parentheses are 95% confidence interval

*p*-values calculated by using the McNemar test to compare the differences between the typical vascular pattern and diagnostic algorithms

*EOB-MRI* gadoxetate acid-enhanced MRI, *ECA-MRI* extracellular contrast agent-enhanced MRI

**Table 4:** Comparison of diagnosis performance between EOB-MRI and ECA-MRI

	EOB-MRI	*p*-value*	ECA-MRI	*p*-value*	*p*-value#
**Training set**					
Typical vascular pattern					
Sensitivity	0.400 (0.293–0.507)	-	0.362 (0.257–0.468)	-	0.678
Specificity	0.984 (0.953–0.999)	-	0.968 (0.924–0.999)	-	> 0.999
Modified diagnosis algorithm					
Sensitivity	0.812 (0.727–0.898)	< 0.001	0.425 (0.317–0.533)	0.063	< 0.001
Specificity	0.903 (0.830–0.977)	0.063	0.984 (0.953–0.999)	> 0.999	0.063
LR-4/5					
Sensitivity	0.425 (0.317–0.533)	0.500	0.450 (0.341–0.559)	0.016	0.839
Specificity	0.984 (0.953–0.999)	> 0.999	0.952 (0.898–0.999)	> 0.999	0.625
**Time-independent test set**					
Typical vascular pattern					
Sensitivity	0.375 (0.181–0.569)	-	0.458 (0.259–0.658)	-	0.774
Specificity	1	-	0.947 (0.847–0.999)	-	> 0.999
Modified diagnosis algorithm					
Sensitivity	0.792 (0.629–0.954)	0.006	0.417 (0.219–0.614)	> 0.999	0.004
Specificity	0.947 (0.847–0.999)	> 0.999	1	> 0.999	> 0.999
LR-4/5					
Sensitivity	0.500 (0.300–0.700)	0.375	0.458 (0.259–0.658)	> 0.999	> 0.999
Specificity	1	> 0.999	0.947 (0.847–0.999)	> 0.999	> 0.999

Values in parentheses are 95% confidence interval

Modified diagnosis algorithm, the combination of T2WI mild-moderate hyperintensity, nonperipheral washout on PVP or TP and restricted diffusion on EOB-MRI, and the combination of T2WI mild-moderate hyperintensity, nonperipheral washout on PVP or DP and restricted diffusion on ECA-MRI

*EOB-MRI* gadoxetate acid-enhanced MRI, *ECA-MRI* extracellular contrast agent-enhanced MRI

* Comparison between the typical vascular pattern and the modified diagnostic algorithm

# Comparison between EOB-MRI and ECA-MRI

In the training set, typical vascular pattern exhibited satisfactory specificity with relatively low sensitivity on both two MRI modalities, and demonstrated no statistical difference in sensitivity (0.400 [95% CI, 0.293–0.507] vs. 0.362 [95% CI, 0.257–0.468], *p* = 0.678) and specificity (0.984 [95% CI, 0.953–0.999] vs. 0.968 [95% CI, 0.924–0.999], *p* > 0.999) between EOB-MRI and ECA-MRI. For EOB-MRI, while ensuring minimal sacrifice of specificity, diagnostic algorithm that combined all three significant MR characteristics (T2WI mild-moderate hyperintensity, nonperipheral washout on PVP or TP and restricted diffusion) exhibited a markedly improved sensitivity relative to the typical vascular pattern (Sensitivity, 0.812 [95% CI, 0.727–0.898] vs. 0.400 [95% CI, 0.293–0.507], *p* < 0.001; Specificity, 0.903 [95% CI, 0.830–0.977] vs. 0.984 [95% CI, 0.953–0.999], *p* = 0.063). For ECA-MRI, among all diagnostic algorithms that maintain high specificity, none showed a significant improvement in sensitivity, including the combination of T2WI mild-moderate hyperintensity, nonperipheral washout on PVP or DP and restricted diffusion. Thereafter, a modified diagnostic algorithm on ECA-MRI was developed by integrating all significant MR characteristics, which showed the highest specificity for diagnosing subcentimeter recurrences. Furthermore, in the time-independent test set, the EOB-MRI-based modified diagnostic algorithm also demonstrated a significant improvement in sensitivity compared with the typical vascular pattern, whereas this improvement was not observed with the ECA-MRI-based modified algorithm.

**Fig. 2:**
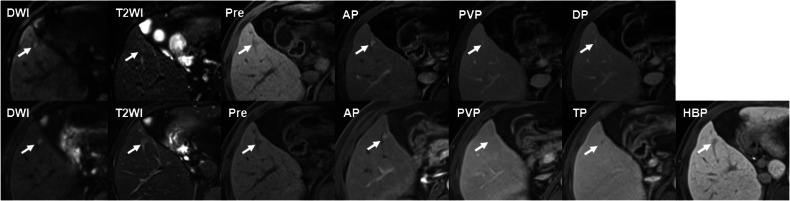
A 54-year-old man with subcentimeter recurrent HCC (0.7 cm, white arrow) underwent both ECA-MRI and EOB-MRI within 8 days. On ECA-MRI, this lesion showed T2WI mild-moderate hyperintensity and restricted diffusion, without nonperipheral washout on PVP or DP, and thus was not diagnosed as recurrent HCC based on ECA-MRI. In EOB-MRI conducted 8 days later, this lesion showed T2WI mild-moderate hyperintensity, restricted diffusion and nonperipheral washout on PVP or TP, thus being diagnosed as recurrent HCC on EOB-MRI-based modified diagnostic algorithm. EOB-MRI, gadoxetate acid-enhanced MRI; ECA-MRI, extracellular contrast agent-enhanced MRI; AP, arterial phase; PVP, portal venous phase; DP, delayed phase; TP, transitional phase; HBP, hepatobiliary phase

The comparison result of the modified diagnostic algorithm based on EOB-MRI and ECA-MRI showed that EOB-MRI-based modified diagnostic algorithm showed significantly superior sensitivity to its ECA-MRI counterpart (0.812 [95% CI, 0.727–0.898] vs. 0.425 [95% CI, 0.317–0.533], *p* < 0.001), with comparable specificity (0.903 [95% CI, 0.830–0.977] vs. 0.984 [95% CI, 0.953–0.999], *p* = 0.063), which was also validated in the time-independent test set (Sensitivity, 0.792 [95% CI, 0.629–0.954] vs. 0.417 [95% CI, 0.219–0.614], *p* = 0.004; Specificity, 0.947 [95% CI, 0.847–0.999] vs. 1.000 [95% CI, -], *p* > 0.999) (Table 4, Fig. 2).

In terms of LI-RADS categorization, its diagnostic performance did not differ significantly between the two imaging modalities. In ECA-MRI, LR-4/5 demonstrated a mildly higher sensitivity than the typical vascular pattern (0.450 [0.341–0.559] vs. 0.362 [0.257–0.468], *p* = 0.016), while specificity remained comparable (0.952 [0.898–0.999] vs. 0.968 [0.924–0.999], *p* > 0.999). In EOB-MRI, however, the diagnostic performance of LR-4/5 showed no statistically significant difference from that of the typical vascular pattern (Sensitivity, 0.425 [95% CI, 0.317–0.533] vs. 0.400 [95% CI, 0.293–0.507], *p* = 0.500; Specificity, 0.984 [95% CI, 0.953–0.999] vs. 0.984 [95% CI, 0.953–0.999], *p* > 0.999).

To address potential selection bias introduced by the imaging sequence, we also performed a subgroup analysis based on the MRI sequence. Among the 153 patients, 114 underwent ECA-MRI prior to EOB-MRI, while 39 patients underwent EOB-MRI first. In both subgroups, the EOB-MRI-based modified algorithm showed significantly higher sensitivity than the ECA-MRI-based algorithm (ECA-first subgroup: 0.797 vs. 0.304, *p* = 0.019; EOB-first subgroup: 0.840 vs. 0.400, *p* = 0.075), with comparable specificity (ECA-first subgroup: 0.934 vs. 1.000, *p* > 0.999; EOB-first subgroup: 0.900 vs. 0.950, *p* = 0.732).

## Discussion

Our study demonstrated that the EOB-MRI-based modified diagnostic algorithm significantly improved sensitivity for diagnosing subcentimeter recurrent HCC over typical vascular patterns (0.812 vs. 0.400, *p* < 0.001), without a substantial loss of specificity. It also outperformed the ECA-MRI-based modified algorithm in sensitivity (0.812 vs. 0.425, *p* < 0.001), with comparable specificity.

Current guidelines define nonrim APHE followed by conventional washout as diagnostic for HCC. However, subcentimeter HCCs often lack these typical vascular features, resulting in limited sensitivity (~ 44–49%) [16, 21]. In alignment with previous studies, merely 40.0% and 36.2% of subcentimeter recurrent HCC in our training set exhibited a typical vascular pattern on EOB-MRI and ECA-MRI, respectively; similar trends were observed in the time-independent test set (37.5% vs. 45.8%). Notably, no significant difference in diagnostic performance of subcentimeter recurrent HCC based on typical vascular pattern was found between the two modalities across both sets, consistent with previous studies [22], emphasizing the need for an optimized diagnostic algorithm with improved sensitivity while maintaining high specificity.

Our study identified T2WI mild-moderate hyperintensity and restricted diffusion as independent predictors of subcentimeter recurrences on both EOB-MRI and ECA-MRI. These MR characteristics, well established in LI-RADS as favoring malignancy, have been shown to enhance diagnostic accuracy for HCC and recurrent HCC [23, 24]. In particular, Kim et al [25] found that T2WI mild-moderate hyperintensity showed the highest sensitivity for distinguishing HCC from benign lesions among hypervascular hepatic lesions ≤ 1 cm. Another study [26] also supported incorporating T2WI for improved detection of HCC ≤ 2 cm, even in lesions lacking nonrim APHE. Similarly, restricted diffusion has been identified as a significant factor for diagnosing subcentimeter HCC [27] and also for subcentimeter recurrent HCC [24]. Therefore, incorporating T2WI mild-to-moderate hyperintensity and restricted diffusion into a modified algorithm in postoperative surveillance has important clinical value for the early detection of recurrent subcentimeter HCC.

Previous studies have shown that small HCCs tend to show nonrim APHE without conventional nonperipheral washout compared to large HCC [28, 29], limiting their detectability. In our training set, only 52.5% and 43.8% of subcentimeter recurrences demonstrated conventional nonperipheral washout on EOB-MRI (PVP) and ECA-MRI (PVP or DP), respectively. On ECA-MRI, although nonperipheral washout on PVP or DP was an independent predictor for subcentimeter recurrent HCC, its low prevalence in recurrences limited the sensitivity improvement of the modified diagnostic algorithm in our study, while high specificity was maintained. Conversely, on EOB-MRI, nonperipheral washout on PVP or TP, instead of conventional nonperipheral washout on PVP, emerged as an independent predictor. Due to early uptake of gadoxetate disodium by hepatocytes on TP, certain lesions such as hemangiomas and dysplastic nodules may exhibit pseudo-washout, which can compromise specificity. Accordingly, current guidelines recommend assessing nonperipheral washout only on PVP on EOB-MRI [8]. However, previous studies suggest that including TP in the washout assessment can improve sensitivity without compromising specificity [14, 15], even in subcentimeter HCC [16]. In our study, EOB-MRI-based modified algorithm—the combination of T2WI mild-moderate hyperintensity, nonperipheral washout on PVP or TP and restricted diffusion—showed significantly improved sensitivity compared with the typical vascular pattern (0.812 vs. 0.400, *p* < 0.001), while also maintaining high specificity.

Interestingly, nonrim APHE was not identified as a significant predictor for diagnosing subcentimeter recurrences in both modalities in our study. Previous studies reported that less than 80% subcentimeter HCC demonstrated nonrim APHE [16, 30], consistent with our study, with 70% and 78.8% of subcentimeter recurrent HCC showing nonrim APHE in the training set on EOB-MRI and ECA-MRI, respectively. As intranodular arterial supply decreases in early hepatocarcinogenesis and later increases [31], the relatively low prevalence of nonrim APHE may be partly attributed to a higher proportion of multicentric recurrences, rather than intrahepatic metastases, among subcentimeter recurrences in our study. This low prevalence may, in turn, limit the diagnostic performance of nonrim APHE in distinguishing subcentimeter recurrences from hypervascular benign lesions.

In clinical practice, CT/MRI are more commonly used than ultrasound for postoperative surveillance of HCC [8]. Prior studies have established that EOB-MRI offers superior diagnostic performance compared to ECA-MRI and CT for HCC detection [32–34]. Our findings further demonstrate that the EOB-MRI-based modified diagnostic algorithm more effectively detected subcentimeter recurrences when compared with its ECA-MRI counterparts. However, early detection of such subcentimeter lesions may raise concerns about potential overtreatment, but such lesions can function as “signaling lesions” that prompt clinicians to adopt a more cautious follow-up strategy. In this context, the EOB-MRI-based modified diagnostic algorithm proposed in our study may serve as a superior screening tool for patients after hepatectomy, as it enables earlier identification of potentially risky lesions and helps overcome the diagnostic limitations of conventional imaging for subcentimeter tumors. This could ultimately reduce diagnostic delays and better preserve the window for effective salvage treatment.

Our study demonstrated limitations. First, as a retrospective single-center study, selection bias was unavoidable. At our institution, the majority of patients are routinely recommended to undergo ECA-MRI for follow-up as it is covered by China’s national insurance, among which a subset receive additional EOB-MRI within a short interval to improve diagnostic confidence. Meanwhile, a portion of patients are also followed up with EOB-MRI as their first imaging modality due to its superior lesion detection capability. This real-world practice results in some patients receiving EOB-MRI after ECA-MRI in our institution, potentially introducing selection bias favoring EOB-MRI. To assess its impact, we compared the diagnostic performance of EOB-MRI and ECA-MRI in the ECA-first subgroup and EOB-first subgroup, respectively, and the EOB-MRI-based modified algorithm showed significantly higher sensitivity than the ECA-MRI-based algorithm in both subgroups, indicating the potential selection bias had minimal impact on the study conclusions. On the other hand, to establish a reliable diagnostic algorithm and ensure diagnostic accuracy for recurrent subcentimeter HCC, we adopted relatively strict exclusion criteria, which resulted in a smaller sample size—particularly in the test set—and may introduce selection bias and limit generalizability. In addition, because our study population consists mainly of HBV-related HCC from a single Chinese tertiary center, the applicability of our findings to populations with other etiologies requires further investigation. Therefore, future large-sample, international, multicenter prospective validation is warranted.

In conclusion, EOB-MRI with a modified algorithm exhibited superior diagnostic performance for subcentimeter recurrent HCC compared with ECA-MRI, providing a more sensitive and reliable imaging strategy for early recurrence identification.

## Supplementary information

ELECTRONIC SUPPLEMENTARY MATERIAL

## Data Availability

The datasets generated or analyzed during this study are available from the corresponding author on reasonable request.

## Abbreviations

APHE: Arterial hyperenhancement
DP: Delayed phase
ECA: Extracellular contrast agent-enhanced
EOB: Gadoxetic acid-enhanced
HBP: Hepatobiliary phase
HCC: Hepatocellular carcinoma
LI-RADS: Liver Imaging Reporting and Data System
PVP: Portal venous phase
TP: Transitional phase
