# Supplementary material for: Performance comparison of gadoxetic and extracellular MRI in diagnosing subcentimeter recurrent HCC after hepatectomy: a modified algorithm
Source: Insights Imaging. 2026 Apr 8;17:97. doi: 10.1186/s13244-026-02266-9 (PMC13062055; doi:10.1186/s13244-026-02266-9)
Supplement: Supplementary file 1 — ELECTRONIC SUPPLEMENTARY MATERIAL [file 13244_2026_2266_MOESM1_ESM.pdf]

Performance comparison of gadoxetic and extracellular MRI in diagnosing subcentimeter recurrent HCC after hepatectomy: a modified algorithm

ELECTRONIC SUPPLEMENTARY MATERIAL

Table S1. Definition of MR characteristics.

MR characteristics	definition
T1WI hypointensity	Intensity in the T1WI unequivocally less, in whole or in part, than liver.
T2WI mild-moderate hyperintensity	Intensity on T2WI mildly or moderately higher than liver and similar to or less than non-iron-overloaded spleen.
Fat deposition	Excess fat within a mass, in whole or in part, relative to adjacent liver.
Nonrim arterial phase hyperenhancement	Nonrim-like enhancement in arterial phase unequivocally greater in<br>whole or in part than liver.
Nonperipheral washout on PVP or DP *	Visually assessed nonperipheral temporal reduction in enhancement in whole or in part relative to composite liver tissue in portal venous phase or delayed phase.
TP hypointensity	Intensity in the transitional phase unequivocally less, in whole or in part, than liver.
Nonperipheral washout on PVP or TP	Visually assessed nonperipheral temporal reduction in enhancement in whole or in part relative to composite liver tissue in portal venous phase or transitional phase.
Enhancing capsule	Smooth, uniform, sharp border around most or all of an observation, unequivocally thicker or more conspicuous than fibrotic tissue around background nodules, and visible as enhancing rim in portal venous phase, or transitional phase.
HBP hypointensity	Intensity in the hepatobiliary phase unequivocally less, in whole or in part, than liver.
Restricted diffusion	Intensity on DWI, not attributable solely to T2 shine-through, unequivocally higher than liver and/or ADC unequivocally lower than liver.

* Nonperipheral washout on PVP for EOB-MRI; nonperipheral washout on PVP or DP for ECA-MRI.

Table S2. MR characteristics of hepatic lesions in EOB-MRI and ECA-MRI.

MR characteristics	EOB-MRI			ECA-MRI			p value [b]
	Recurrences	Non-recurrences	p value [a]	Recurrences	Non-recurrence	p value [a]	
**Training set**							
T1WI hypointensity	96.3% (77/80)	74.2% (46/62)	<0.001	88.8% (71/80)	66.1% (41/62)	0.001	0.035
T2WI mild-moderate hyperintensity	93.8% (75/80)	41.9% (26/62)	<0.001	92.5% (74/80)	46.8% (29/62)	<0.001	0.851
Fat deposition	11.3% (9/80)	6.5% (4/62)	0.325	10.0% (8/80)	4.8% (3/62)	0.348	0.727
Nonrim APHE	70.0% (56/80)	48.4% (30/62)	0.009	78.8% (63/80)	53.2% (33/62)	0.001	0.143
Nonperipheral washout on PVP or DP [*]	52.5% (42/80)	6.5% (4/62)	<0.001	43.8% (35/80)	12.9% (8/62)	<0.001	0.743
TP hypointensity	90.0% (72/80)	43.5% (27/62)	<0.001	-	-	-	-
Nonperipheral washout on PVP or TP	88.8% (71/80)	27.4% (17/62)	<0.001	-	-	-	-
Enhancing capsule	8.8% (7/80)	1.6% (1/62)	0.138	16.3% (13/80)	1.6% (1/62)	0.004	0.146
HBP hypointensity	98.8% (79/80)	80.6% (50/62)	<0.001	-	-	-	-
Restricted diffusion	92.5% (74/80)	38.7% (24/62)	<0.001	90.0% (72/80)	43.5% (27/62)	<0.001	>0.999
LI-RADS categorization			<0.001			<0.001	0.572
LR-3	57.5% (46/80)	98.4% (61/62)		55.0% (44/80)	95.2% (59/62)		
LR-4/5	42.5% (34/80)	1.6% (1/62)		45.0% (36/80)	4.8% (3/62)		
**Time-independent test set**							
T1WI hypointensity	100% (24/24)	57.9% (11/19)	0.001	83.3% (20/24)	57.9% (11/19)	0.065	0.289
T2WI mild-moderate hyperintensity	95.8% (23/24)	31.6% (6/19)	<0.001	83.3% (20/24)	31.6% (6/19)	0.001	0.508
Fat deposition	8.3% (2/24)	5.3% (1/19)	0.695	8.3% (2/24)	5.3% (1/19)	0.695	>0.999
Nonrim APHE	79.2% (19/24)	31.6% (6/19)	0.002	87.5% (21/24)	68.4% (13/19)	0.153	0.049
Nonperipheral washout on PVP or DP	58.3% (14/24)	5.3% (1/19)	<0.001	45.8% (11/24)	15.8% (3/19)	0.037	>0.999
TP hypointensity	87.5% (21/24)	42.1% (8/19)	0.002	-	-	-	-
Nonperipheral washout on PVP or TP	83.3% (20/24)	15.8% (3/19)	<0.001	-	-	-	-

Enhancing capsule	33.3% (8/24)	5.3% (1/19)	0.055	33.3% (8/24)	5.3% (1/19)	0.055	>0.999
HBP hypointensity	95.8% (23/24)	73.7% (14/19)	0.072	-	-	-	-
Restricted diffusion	83.3% (20/24)	26.3% (5/19)	<0.001	75.0% (18/24)	42.1% (8/19)	0.028	>0.999
LI-RADS categorization			<0.001			0.003	>0.999
LR-3	50.0% (12/24)	100.0% (19/19)		54.2% (13/24)	94.7% (18/19)		
LR-4/5	50.0% (12/24)	0% (0/19)		45.8% (11/24)	5.3% (1/19)		

Note. – Values are percentage (number).

EOB-MRI, gadoxetate acid-enhanced MRI; ECA-MRI, extracellular contrast agent enhanced MRI.

[a] p values calculated by using chi-square test or Fisher's exact test to compare the differences between recurrences and non-recurrences.

[b] p values calculated by using McNemar test to compare the differences between EOB-MRI and ECA-MRI.

[*] Nonperipheral washout on PVP for EOB-MRI; nonperipheral washout on PVP or DP for ECA-MRI.
